# CXC chemokines and antimicrobial peptides in rhinovirus‐induced experimental asthma exacerbations

**DOI:** 10.1111/cea.12313

**Published:** 2014-06-23

**Authors:** G. Rohde, S. D. Message, J. J. Haas, T. Kebadze, H. Parker, V. Laza‐Stanca, M. R. Khaitov, O. M. Kon, L. A. Stanciu, P. Mallia, M. R. Edwards, S. L. Johnston

**Affiliations:** ^1^ Department of Respiratory Medicine National Heart and Lung Institute MRC and Asthma UK Centre in Allergic Mechanisms of Asthma & Centre for Respiratory Infection Imperial College London London UK; ^2^ Department of Respiratory Medicine Maastricht University Medical Centre+ Maastricht The Netherlands; ^3^ Imperial College Healthcare NHS Trust London UK; ^4^ State National Center Institute of Immunology Federal Medicobiological Agency of Russia Moscow Russia

**Keywords:** airway epithelium, infection control, innate immunity, neutrophil biology, respiratory infection

## Abstract

**Rationale:**

Rhinoviruses (RVs) are the major triggers of asthma exacerbations. We have shown previously that lower respiratory tract symptoms, airflow obstruction, and neutrophilic airway inflammation were increased in experimental RV‐induced asthma exacerbations.

**Objectives:**

We hypothesized that neutrophil‐related CXC chemokines and antimicrobial peptides are increased and related to clinical, virologic, and pathologic outcomes in RV‐induced exacerbations of asthma.

**Methods:**

Protein levels of antimicrobial peptides (SLPI, HNP 1–3, elafin, and LL‐37) and neutrophil chemokines (CXCL1/GRO‐α, CXCL2/GRO‐β, CXCL5/ENA‐78, CXCL6/GCP‐2, CXCL7/NAP‐2, and CXCL8/IL‐8) were determined in bronchoalveolar lavage (BAL) fluid of 10 asthmatics and 15 normal controls taken before, at day four during and 6 weeks post‐experimental infection.

**Results:**

BAL HNP 1–3 and Elafin were higher, CXCL7/NAP‐2 was lower in asthmatics compared with controls at day 4 (*P* = 0.035, *P* = 0.048, and *P* = 0.025, respectively). BAL HNP 1–3 and CXCL8/IL‐8 were increased during infection (*P* = 0.003 and *P* = 0.011, respectively). There was a trend to increased BAL neutrophils at day 4 compared with baseline (*P* = 0.076). BAL HNP 1–3 was positively correlated with BAL neutrophil numbers at day 4. There were no correlations between clinical parameters and HNP1–3 or IL‐8 levels.

**Conclusions:**

We propose that RV infection in asthma leads to increased release of CXCL8/IL‐8, attracting neutrophils into the airways where they release HNP 1–3, which further enhances airway neutrophilia. Strategies to inhibit CXCL8/IL‐8 may be useful in treatment of virus‐induced asthma exacerbations.

## Introduction

Patients with atopic asthma are more susceptible to lower respiratory tract (LRT) infections and have more severe and longer‐lasting rhinovirus (RV)‐induced LRT symptoms than healthy individuals 1. Virus infections of the respiratory tract are frequently associated with asthma exacerbations, with RVs as the predominant viruses 2, 3. RVs directly infect the lower airways 4 resulting in increased lower respiratory symptoms, reductions in lung function, bronchial inflammation, and augmented airway hyperresponsiveness in asthmatic compared with normal subjects 5.

Neutrophils are major effector cells in defence against invading pathogens 6, and their number has been shown to increase during RV infection in both experimental models 5, 7 and naturally occurring asthma exacerbations 8. Antimicrobial peptides of the defensin or cathelicidin families comprise a significant part of the neutrophilic armamentarium against these pathogens 6. The α‐defensins (HNP 1–3) are stored in primary (azurophil) neutrophil granules and constitute 30–50% of the total protein of these organelles 9. It has been hypothesized that human rhinovirus infections should increase levels of α‐defensins in the airways 10, as they lead to marked neutrophil infiltration and degranulation in the airways 11 which are associated with clinical severity of virus‐induced asthma 5, 12. However, there have been no reports directly measuring defensins in the airways of subjects with virus‐induced asthma so far.

The human cathelicidin LL‐37 is also released by neutrophils upon inflammatory stimulation and has potent bactericidal activity 13. SLPI is another antimicrobial peptide produced by neutrophils (also by alveolar macrophages and epithelial cells) which may play a role in acute exacerbations of asthma. Their role in virus‐induced asthma is unknown.

Neutrophils are attracted to the airways and are activated mainly by the CXC chemokines CXCL1/GRO‐α, CXCL2/GRO‐β, CXCL5/ENA‐78, CXCL6/GCP‐2, CXCL7/NAP‐2, and CXCL8/IL‐8. Some of these (CXCL1/GRO‐α, CXCL2/GRO‐β, and CXCL6/GCP‐2) also have antimicrobial properties, while it has also been shown that elafin, another antimicrobial peptide expressed by alveolar macrophages and epithelial cells, is also chemotactic for neutrophils 14.

Against this background, we hypothesized that antimicrobial peptides are induced by RV infections in the lower airways *in vivo*. To test this hypothesis and to clarify whether this possible induction is related to airway neutrophilia and the expression of CXC chemokines, we analysed the expression of neutrophil antimicrobial peptides and CXC chemokines in BAL fluid of subjects with RV‐induced experimental asthma exacerbations.

Some of the results of this study have been previously reported in abstract form 15.

## Subjects, materials and methods

### Subjects

The study design and the clinical and lower airway inflammation data of the patients investigated have been recently published in detail 5. Briefly, two different groups were studied. The first group consisted of outpatients with mild atopic asthma; the second group were healthy non‐atopic individuals. Clinical and atopic status were defined by questionnaire, skin prick testing, serum IgE, and lung function testing including PEF, forced expiratory volume in 1 s (FEV_1_), forced vital capacity (FVC), and histamine challenge performed according to guidelines 16. The asthmatic group had a concentration of histamine causing a 20% reduction in FEV_1_ (PC_20_) < 8 mg/mL, the normal group > 8 mg/mL. Normal subjects were taking no medication; asthmatics inhaled short‐acting β2‐agonists only. None of the asthmatic patients were given any inhaled or oral/systemic steroid at any time point in the study. Subjects were free of common cold symptoms for 6 weeks before commencing the study. All were non‐smokers.

Bronchoalveolar lavage (BAL) sampling was carried out at baseline (2 weeks prior to virus inoculation), on day 4 after inoculation (acute infection) and at 6 weeks after inoculation (convalescent). Diaries were kept to record symptoms and home lung function throughout the study. All subjects gave written informed consent, and the study was approved by the St Mary's Research Ethics Committee, St Mary's Hospital, London, UK.

All subjects were seronegative (neutralizing antibody titre < 1 : 2) for RV16 at screening and on repeat serology performed on day 0 prior to inoculation, and all subjects were negative to a PCR panel for respiratory viruses (adenoviruses, coronaviruses 229E and OC43, human metapneumovirus, influenza AH1/AH3/B, other picornaviruses, parainfluenza viruses 1–3, and respiratory syncytial virus) and *Mycoplasma* and *Chlamydophila pneumoniae* in nasal lavage at baseline 5.

### Experimental RV‐16 infection

Experimental infection was induced using 10 000 TCID_50_ RV16 17 on day 0, with a DeVillbiss 286 atomizer as described 5. Following inoculation, subjects returned home.

### Clinical assessment of RV16 infection

Clinical effects of RV16 infection were recorded using daily diary cards enabling the calculation of a peak cold score, a total cold score (total over the 2 week period post‐inoculation), peak and total chest scores (all corrected for baseline symptoms and effect of bronchoscopy), lung function testing by home spirometry (microDL, MicroMedical, Carefusion, Basingstoke, UK), and histamine challenge were performed as described 5.

### Processing of BAL

Bronchoalveolar lavage was collected in a single plastic chamber and transferred immediately to polypropylene tubes on ice for transport to the laboratory.

An aliquot of BAL fluid was stored unprocessed at −80°C for analysis of virus load by PCR. The remaining BAL fluid was centrifuged, and BAL fluid was stored in aliquots at −80°C. The BAL cell pellet was used for cytospin preparations for differential cell counting as described 5.

### Confirmation of RV16 infection

Rhinovirus infection was confirmed in all subjects using PCR, by culture or by serology as described 5.

Virus load was determined in nasal lavage and the unprocessed BAL aliquot by quantitative PCR as described 5.

### Quantification of antimicrobial peptides in BAL fluid

In BAL fluid, SLPI levels were assessed by enzyme‐linked immunosorbent assay (ELISA) using a commercially available kit (R&D Systems, Abingdon, UK) with a sensitivity of < 25 pg/mL. Samples were diluted 1 : 200. HNP 1–3 levels were measured by ELISA, using a commercially available kit (Hycult Biotechnology, Uden, the Netherlands) with a sensitivity of < 156 pg/mL. Samples were diluted 1 : 100. Elafin and human LL‐37 were assessed by ELISA kits from Cambridge Bioscience, UK, with sensitivities of < 878 pg/mL and < 0.1 ng/mL, respectively.

### Chemokine analysis in BAL fluid

CXCL8/IL‐8, CXCL5/ENA‐78, and CXCL1/GRO‐α levels in BAL fluid were assessed by Luminex analysis (Biosource) on the Luminex TM 100 system with sensitivities of < 3, < 5, and < 5 pg/mL, respectively. CXCL6/GCP‐2 and CXCL7/NAP‐2 were analysed by ELISA using commercially available kits (R&D systems) with sensitivities of < 7.8 pg/mL as well as CXCL2/GRO‐β (Antigenix America Inc, Huntington Station, NY, USA) with a sensitivity of < 10 pg/mL.

### Statistical analysis

All data were checked for normal distribution by Kolmogorov–Smirnov test. Normally distributed data are presented as mean and standard deviation, whereas non‐normally distributed data are presented as median and interquartile range.

Differences between normal and asthmatic groups were analysed using unpaired *t*‐tests for normally distributed data and Mann–Whitney test for non‐normally distributed data.

For discrete variables, frequencies were reported and compared by chi‐square test or Fisher's exact test as appropriate. The Yates correction procedure was applied to all comparisons.

Differences during infection from baseline and convalescence were analysed using one‐way repeated‐measures anova for normally distributed data. Sphericity was assessed by Mauchly's test. If the assumption of sphericity was violated, degrees of freedom were corrected using Greenhouse–Geisser correction for ε < 0.75 or Huynh–Feldt correction for ε > 0.75, respectively.

In the case of significant differences, post hoc tests (Bonferroni correction) were performed. In case of non‐ normally distributed data, Friedman's test was used and, if significant, *post hoc* tests (Wilcoxon) were performed.

Correlations for normally distributed variables were examined using Pearson's correlation coefficient, for non‐normally distributed variables using Spearman's correlation coefficient, and the respective two‐tailed significance was reported.

All significance levels were set to 5%. Data were analysed and processed using graphpad prism 4.0 (GraphPad Software, Inc., La Jolla, CA, USA) and spss 18.0 (International Business Machines Corp., Armonk, NY, USA).

## Results

The study design, the analysis of clinical characteristics and the clinical response to experimental viral infection together with extensive data on the effect on the Th1/Th2 immune response have been reported 5. However, here, we present a completely new analysis of data from those 10 atopic asthmatics and 15 non‐atopic normal controls that entirely completed the study.

### Subjects

Baseline characteristics of all recruited subjects (11 asthmatics and 17 controls) have been reported by Message et al. 5 recently. One asthmatic and two normal volunteers did not continue after the baseline phase. The clinical characteristics of the 25 subjects that completed the study and who underwent the chemokine and anti‐microbial peptide analyses reported in the present study are summarized in Table 1.

**Table 1:** Clinical characteristics

	Asthmatics (*n* = 10)	Controls (*n* = 15)	Statistics[Link], [Link] (*P*)
Age (years)	22.0 (2.8)	26.9 (8.9)	0.060
Gender (F = female, M = male)	8 F/2 M	7 F/8 M	0.211
Baseline FEV_1_ (% predicted)	106.3 (14.0)	103.3 (13.7)	0.596
Total IgE (IU/mL)	249.3 (156.4)	26.6 (31.6)	**0.001**
Skin prick test (positive/negative)	9/1	0/15	**< 0.001**

Data are presented as mean and (standard deviation) or as absolute numbers (gender and skin prick test).

FEV_1_, forced volume in 1 s; IgE, immunoglobulin E.

*Independent‐samples *t*‐test was used for continuous and chi‐square test for categorical variables.

*Significantly different results are printed in bold.

There were no significant differences between groups concerning age, gender, and baseline FEV_1_. Features of allergic sensitization were only expressed in the asthmatic group.

We reported before that asthmatic patients showed significantly higher total chest symptom score, significantly higher maximum falls in FEV_1_ and PEF, and significantly lower PC10 values at baseline, day 6, and week 6 compared with healthy controls 5. Lung function impairment induced by RV infection was correlated with increased neutrophils in BAL of asthmatics suggesting a role for PMNs in RV‐induced exacerbations of asthma 5. There were no significant differences in virus load in upper and lower airway samples between the two groups.

### Airway levels of antimicrobial peptides and neutrophil chemoattractant chemokines

Results are summarized in Table 2.

**Table 2:** Multivariate analysis of cells and levels of soluble mediators in bronchoalveolar lavage (BAL)

	Asthmatics				Controls				
	Baseline	Day 4	Week 6	Repeated‐measures anova (*P*)	Baseline	Day 4	Week 6	Repeated‐measures anova (*P*)	Between groups univariate analysis (*P*)
Total cells (× 10^6^/L)	94.7 (47.7)	127.0 (40.1)	117.8 (38.1)	0.179	102.8 (29.8)	104.3 (39.2)	125.0 (45.6)	0.067	ns
Neutrophils (× 10^6^/L)	1.3 (1.0)	7.5 (10.9)	2.2 (1.2)	0.077	1.4 (0.9)	1.9 (2.1)	1.4 (1.0)	0.359	ns
Antimicrobial peptides:									
SLPI (ng/mL)	169.9 (113.7)	304.2 (265.1)	153.7 (66.9)	0.144	157.2 (82.4)	165.1 (64.5)	160.3 (101.1)	0.943	ns
HNP 1–3 (ng/mL)	**0.9 (0.8)**	**1.4 (0.7)**	**0.7 (0.6)**	**0.003**	0.4 (0.4)	*0.7 (0.4)*	0.5 (0.4)	0.160	*0.035* at day 4
LL 37 (ng/mL)	0.1 (0.1)	0.1 (0.0)	0.1 (0.1)	0.768	0.2 (0.1)	0.2 (0.1)	0.1 (0.1)	0.470	ns
Elafin (ng/mL)	1465.0 (1826.8)	*1595.6 (791.4)*	1154.1 (193.8)	0.736	709.7 (479.1–1250.8)	*823.7 (587.4*–*1302.1)*	720.8 (320.5–1523.1)	0.880^a^	*0.048* at day 4
CXC chemokines:									
CXCL1/GRO‐α (pg/mL)	654.8 (328.3)	805.0 (340.3)	520.3 (244.7)	0.060	693.2 (193.7)	655.4 (226.2)	649.1 (225.2)	0.733	ns
CXCL2/GRO‐β (pg/mL)	290.4 (75.0)	300.5 (158.7)	370.4 (125.4)	0.133	317.6 (115.9)	327.8 (102.5)	305.8 (75.0)	0.808	ns
CXCL5/ENA‐78 (pg/mL)	14.5 (4.4)	29.4 (16.3)	20.1 (22.6)	0.083	11.8 (9.2–16.1)	14.5 (12.0–31.2)	11.4 (9.7–26.2)	0.199^a^	ns
CXCL6/GCP‐2 (pg/mL)	2207.2 (1117.7)	2604.4 (1737.8)	2621.9 (1707.3)	0.708	3128.3 (1074.0)	3212.1 (1295.8)	3302.1 (1519.1)	0.877	ns
CXCL7/NAP‐2 (pg/mL)	99.3 (135.2)	*45.8 (16.2)*	54.2 (27.5)	0.392	54.7 (39.8–280.7)	*102.7 (45.2–213.9)*	71.2 (47.4–90.8)	0.232^a^	*0.025* at day 4
CXCL8/IL‐8 (pg/mL)	**13.4 (10.9)**	**94.2 (68.3)**	**17.2 (22.1)**	**0.011**	*25.0 (15.2)*	50.1 (59.9)	36.5 (55.4)	0.183	*0.038* at baseline

ns, not significant.

*P*‐values showing statistically significant differences within groups are marked in bold. Numbers in brackets represent standard deviation. *P*‐values showing statistically significant differences between groups are marked in italic.

a Data were non‐normally distributed and hence analysed with Friedman's test, and numbers in brackets here refer to the range of data.

**Figure 1:**
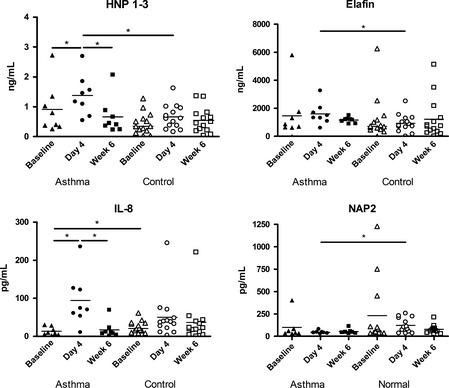
Differences in CXC chemokines and HNP 1–3 in asthmatics (closed symbols) and normals (open symbols) at baseline (triangle), Day 4 (circle) or Week 6 (rectangle). Median values are illustrated by a horizontal bar for each group. Significant differences between groups are indicated by horizontal lines above. Stars indicate significance levels, *P < 0.05.

To determine differences in mediator release between normal and asthmatics subjects before, during, and after RV infection, a univariate analysis between groups was performed. This showed that BAL CXCL8/IL‐8 was the only parameter significantly different at baseline. Interestingly, it was higher in the control group compared with asthmatics (25.0 (15.2) vs. 13.4 (10.9) pg/mL, *P* = 0.038, Fig. 1). Four days after infection, BAL HNP 1–3 and elafin were significantly higher in asthmatics compared with controls (1.4 (0.7) vs. 0.7 (0.4) ng/mL, *P* = 0.035, 1595.6 (791.4) vs. 823.7 (587.4–1302.1) ng/mL, *P* = 0.048, respectively, Fig. 1), while BAL CXCL7/NAP‐2 was significantly higher in controls compared with asthmatics (102.7 (45.2–213.9) vs. 45.8 (16.2), *P* = 0.025, Fig. 1).

Repeated‐measures multivariate analysis showed significant differences only in asthmatic subjects. BAL HNP 1–3 and CXCL8/IL‐8 were significantly increased at day 4 compared with baseline (Fig. 1 and Table 2). BAL HNP 1–3 and CXCL8/IL‐8 only were also significantly increased at day 4 compared with week 6 in asthmatic subjects (Fig. 1 and Table 2). There was a trend to increased BAL neutrophils at day 4 compared with baseline in asthmatic subjects (*P* = 0.076).

We also measured BAL IL‐6, but no significant differences were observed, neither within groups at the different time points nor between asthmatics and controls at any time point (data not shown).

### Relationship between BAL neutrophils, soluble mediators, virus load, and clinical parameters

**Figure 2:**
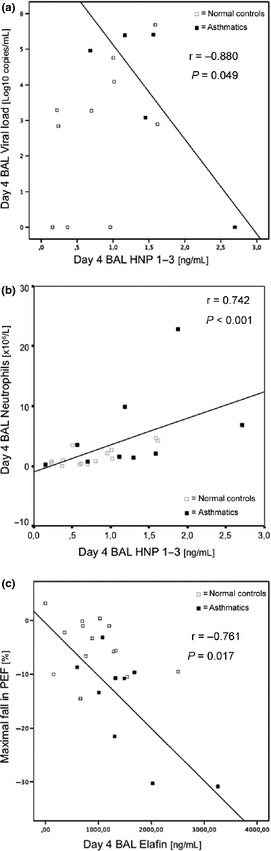
(a) The relationship between bronchoalveolar lavage (BAL) HNP 1–3 levels and BAL viral load at day 4 post‐infection was investigated in the two subject groups. In the asthmatic group (■), there was a significant inverse correlation between HNP 1–3 and BAL viral load at day 4 post‐infection, which was not present in the normal group (□). (b) The relationship between BAL HNP 1–3 levels and BAL neutrophils at day 4 post‐infection was investigated in the two subject groups. In the asthmatic group (■), there was a significant correlation between HNP 1–3 and BAL neutrophils at day 4 post‐infection. The same relationship was observed in the normal group (□). (c) The relationship between BAL elafin levels at day 4 post‐infection and peak flow maximal fall was investigated in the two subject groups. In the asthmatic group (■), there was a significant inverse correlation between BAL elafin and peak flow maximal fall at day 4 post‐infection, which was not present in the normal group (□).

BAL HNP 1–3 measured at baseline was negatively correlated with BAL viral load (*r* = −0.880, *P* = 0.049) in asthmatics only (Fig. 2a). BAL viral load was available in 5 asthmatic subjects only. Unfortunately, the other 5 samples got lost during a liquid nitrogen thawing over Christmas/New Year and were not available for analysis. BAL HNP 1–3 at baseline was correlated with BAL CXCL8/IL‐8 at baseline in asthmatics only (*r* = 0.753, *P* = 0.031). BAL HNP 1–3 was the only parameter to be positively correlated with relative BAL neutrophil numbers at day 4 post‐infection (in all subjects; Fig. 2b). At week 6, BAL HNP 1–3 was also correlated with BAL CXCL8/IL‐8 (*r* = 0.469, *P* = 0.028) in all subjects.

BAL CXCL8/IL‐8 and CXCL1/GRO‐α levels at day 4 post‐infection were correlated with peak nasal lavage virus load (*r* = 0.721, *P* = 0.044, and *r* = 0.738, *P* = 0.037, respectively) in asthmatics. BAL CXCL8/IL‐8 at week 6 was correlated with BAL neutrophils (in% of all non‐epithelial cells; *r* = 0.496, *P* = 0.016) in all subjects.

There were no correlations between clinical parameters (FEV_1_ or PEF) and HNP1–3 or IL‐8 levels.

### Relationship between Elafin and Peak Expiratory Flow

Elafin levels at day 4 post‐infection were inversely related to maximal falls in PEF (*r* = −0.761, *P* = 0.017) in asthmatics (Fig. 2c).

## Discussion

We have investigated the effect of RV infection on the expression of CXC chemokines and antimicrobial peptides in a human experimental model of RV‐induced asthma exacerbation. We show, in accordance with Turner et al. 18, that the neutrophil‐attracting chemokine CXCL8/IL‐8 is significantly increased in asthmatics compared with normal controls. BAL neutrophils tended to be increased in asthmatics at day 4 compared with normal controls and their number was related to HNP 1–3 levels. Significantly higher levels of the antimicrobial peptide HNP 1–3 were released into the airways of asthmatic patients compared with normal controls during infection.

Respiratory infections are the main triggers of asthma exacerbations. Respiratory viruses are the most frequent pathogens, and human RVs are most frequently detected 2, 3. It has been shown that during naturally occurring virus‐induced asthma exacerbations, neutrophils are recruited into the airways as part of the immune defence 8. The influx of neutrophils correlates with symptoms and parameters of airways obstruction such as FEV_1_ 19. Accordingly, we observed in our experimental model higher values of BAL neutrophils at day 4 after intranasal experimental infection. However, these changes were moderate, probably due to the small number of patients and the rather mild severity of the induced asthma exacerbations. Recruitment into this intensive and burdensome study was difficult resulting in small numbers of patients. Moreover, also due to ethical constraints, experimental exacerbations had to be mild in character. The results presented are thus also consequences of these requirements. Symptoms and reductions in FEV_1_ were significantly greater in asthmatics compared with controls as previously reported 5. It has been shown before that 4 days after experimental RV infection, the inflammatory response of the upper airways is increased which is associated with increased symptoms and airways obstruction in asthmatics 20.

We report that the increase in neutrophils is associated with higher HNP 1–3 levels. This suggests that neutrophils could be the major source of HNP 1–3. To our knowledge, the only other cell type for which HNP 1–3 expression has been shown is γδ‐CD 8 cells in blood 21. Hence, we do not expect that there are any other relevant cellular sources of HNP 1–3 than neutrophils in BAL. In favour of this is also the fact that CXCL8/IL‐8 was the only chemokine significantly increased at day 4 in asthmatics. It has been shown that HNP 1–3 can induce CXCL8/IL‐8 22, which may explain to a certain degree the significantly higher levels observed at day 4 5. Significantly higher levels of CXCL8/IL‐8 and a trend for higher CXCL1/GRO‐α in BAL at day 4 were related to high virus load measured in nasal lavage. This may be a result of increased induction of CXCL8/IL‐8 and CXCL1/GRO‐α in asthmatics by RVs. It has been shown *in vitro* that RV infection of human respiratory epithelial cell line significantly increases CXCL8/IL‐8 23. Moreover, it has been demonstrated that the intramuscular injection of synthetic HNP1 induces the transcript expression of genes encoding both pro‐inflammatory cytokines (IL‐1beta and TNF‐alpha) and the chemokine CXCL8/IL‐8. Furthermore, HNP1 showed chemotactic capacity towards leucocytes 24. These findings suggest that RV infection induces CXCL8/IL‐8, which has chemotactic activity towards neutrophils, thereby increasing neutrophil numbers in the airway which release HNP 1–3 which has properties that will further enhance neutrophilia.

However, it has to be acknowledged that it is possible that increased defensin expression could also be an epiphenomenon of neutrophil activation and that other mechanisms, such as release of reactive oxygen species or other pro‐inflammatory mediators, may at least also contribute to drive an asthma exacerbation.

All CXC chemokines investigated here are chemoattractant for neutrophils, the major effector cells during asthma exacerbation and viral airway infection. Interestingly, they signal through a common receptor (CXCR2) 25. CXCR2 is required for RV induction of neutrophilic airway inflammation and development of airway hyperresponsiveness as recently demonstrated in a mouse model 26. Hence, CXCR2 could be an interesting target for therapy in RV‐induced asthma exacerbations 27. Specific CXCR1/2 receptor antagonists are already in clinical development 28.

Why might RV infection lead to increased expression of human neutrophil peptides? Antimicrobial peptides such as HNP 1–3 are important effector molecules of neutrophils. It was suggested that α‐defensins (HNP 1–3) cannot directly inactivate non‐enveloped viruses such as RVs 10. However, recent research showed that this is not completely true. It was shown that human α‐defensins (HD‐5) can block adenovirus uncoating 29. Moreover, it is known that HNP 1–3 are potent antagonists of infection by both cutaneous and mucosal papillomavirus types by blocking virion escape from endocytic vesicles 30. Thus, HNP 1–3 do have direct antiviral properties against non‐enveloped viruses and might also have direct antiviral properties against RV infection. In addition, HNP 1–3 might have indirect antiviral effects. They have recently been shown to inhibit HIV‐1 replication even when added 12 h post‐infection 31. Moreover, it was demonstrated that HNP1 can affect the ability of adenoviruses to infect epithelial cells 32. α‐defensins have chemoattractant properties towards both CD8^+^ and CD4^+^/CD45RA^+^ T cells 33. Increased levels of α‐defensins during viral infection could therefore recruit both CD4^+^ and CD8^+^ cells to the airways. This may enhance antiviral immunity as it has been shown in a variety of murine models that HNPs enhance antigen‐specific humoral and cellular immunity 34, 35, 36. However, one has to bear in mind as laid out above that increased defensin expression could also be an epiphenomenon of airways neutrophila which is considered to contribute to asthma exacerbations.

Elafin levels were significantly higher in asthmatics at day 4 compared with normal controls and were 1000 times higher than those of HNP 1–3. However, elafin levels were not significantly increased at day 4 compared with baseline or convalescence. Elafin levels at day 4 post‐infection were inversely related to maximal falls in PEF. This correlation in the absence of a significant increase in response to RV infection (probably due to low subject numbers) must be interpreted with caution. It might suggest that insufficient expression of this molecule might lead to more pronounced functional consequences of RV infection in asthmatics. However, this has to be supported in further experimental and/or clinical studies.

BAL CXCL7/NAP‐2 levels were significantly lower in asthmatics at day 4 compared with controls. It has been shown in a ferret model using microarray analysis that infection with 2009‐H1N1 A/California/07 induced the expression of multiple chemokines including CCL2/MCP‐1, CCL8/MCP‐2, CCL13/MCP‐4, CCL19/ELC, CXCL7/NAP‐2, and CXCL10/IP‐10 37. A recent study found that increased CCL5/RANTES and CXCL7/NAP‐2 expression was associated with neutrophil activation in severe stable COPD. It seems that CXCL7/NAP‐2 plays a role in the local innate immune response and that dysregulation of the expression of this molecule might result in neutrophil dysfunction 38. Clearly, this hypothesis has to be investigated further.

This study has strengths and weaknesses. The major strength of this study is the study design. Experimental RV infection in humans provides an excellent model of virus‐induced asthma under controlled conditions including application of a standard dose of a single virus serotype and standardized clinical data collection. Invasive sampling can be carried out under controlled conditions repeatedly and at accurately defined time points. However, this elaborate study design is extremely labour‐intensive which accounts for limitation of number of subjects that can be included in such a study. Thus, subject numbers have to be small. For safety reasons, only mild asthmatics could be included into the study. This limits the ability to study more severe forms of asthma. Another important aspect is that BAL sampling time points had to be limited to 3 due to the invasive character of this investigation, and it seems possible that the time points chosen (baseline, 4 days and 6 weeks after experimental infection) do not correspond to the peak changes in CXC chemokine and/or AMP expression. Moreover, the analysis of soluble markers in respiratory secretions is complex because of dilution, modification, and degradation. Nevertheless, we found significant differences between asthmatics and normal controls which results from meticulous patient characterization before inclusion. Regarding *in vitro* findings and preliminary *in vivo* data, our results deliver direct evidence that RV infections increase levels of α‐defensins in the airways. This has been assumed as RV infections lead to marked neutrophil infiltration and degranulation in the airways 11, 39. This finding is of possible importance as neutrophil degranulation is associated with clinical severity of virus‐induced asthma 12.

## Conclusion

This is the first study showing increased expression of neutrophil antimicrobial peptides in a well‐defined human model of experimental rhinoviral infection of asthmatics. We propose that RV infection in asthma leads to increased release of CXCL8/IL‐8 thereby attracting neutrophils into the airways where they release HNP 1–3 which further enhances airway neutrophilia. Further studies are warranted to better understand the role and importance of these cells and molecules in asthma exacerbations in order to identify possible new targets for therapy.

## Financial support

European Respiratory Society (Fellowship number 243 to GR), Medical Research Council, UK Clinical Research Fellowship (SDM), British Medical Association HC Roscoe Research Grant (SDM and VLS), Asthma UK (grant numbers 02/027 and 05/067), British Lung Foundation/Severin Wunderman Family Foundation (Programme Grant 00/02), Wellcome Trust (grant no. 063717 and 083567/Z/07/Z), an National Institute for Health Research (NIHR) Clinical Lectureship (PM) and by the NIHR Biomedical Research Centre funding scheme.

## Author's contributions

GR was involved in the hypothesis delineation, the analysis and interpretation of data, and wrote the manuscript. SDM was involved in the conception, hypotheses delineation, and design of the study, the analysis and interpretation of data and had substantial involvement in the revision of the manuscript prior to submission. JH, TK, HP, and VLS were involved in the acquisition of the data and the analysis and interpretation of data. MRK, OMK, LAS, PM, and MRE were involved in the conception, hypotheses delineation, and design of the study, acquisition of the data and the analysis and substantially revised the manuscript prior to submission. SLJ was involved in the conception, hypotheses delineation, and design of the study, the analysis and interpretation of data and in writing and revising the article prior to submission.

## Conflict of interest

All authors declare to have no real or perceived conflict of interest related to the submitted work.
